# Accessible Home Environments for People with Functional Limitations: A Systematic Review

**DOI:** 10.3390/ijerph13080826

**Published:** 2016-08-17

**Authors:** Hea Young Cho, Malcolm MacLachlan, Michael Clarke, Hasheem Mannan

**Affiliations:** 1 Centre for Global Health, Trinity College Dublin, 7-9 Leinster Street South, Dublin 2, Ireland; choh@tcd.ie; 2 School of Psychology, Trinity College Dublin, College Green, Dublin 2, Ireland; 3 Centre for Rehabilitation Studies, Stellenbosch University, Private Bag X1, Matieland, Stellenbosch 7602, South Africa; 4 Olomouc University Social Health Institute, Palacky University Olomouc, Olomouc 77111, Czech Republic; 5 Northern Ireland Network for Trials Methodology Research, Centre for Public Health, Queen’s University Belfast, Belfast BT126BA, UK; m.clarke@qub.ac.uk; 6 School of Nursing, Midwifery & Health Systems, University College Dublin, Belfield, Dublin 4, Ireland; Hasheem.Mannan@ucd.ie

**Keywords:** International Classification of Functioning, disability and health, architectural accessibility, activities of daily living

## Abstract

The aim of this review is to evaluate the health and social effects of accessible home environments for people with functional limitations, in order to provide evidence to promote well-informed decision making for policy guideline development and choices about public health interventions. MEDLINE and nine other electronic databases were searched between December 2014 and January 2015, for articles published since 2004. All study types were included in this review. Two reviewers independently screened 12,544 record titles or titles and abstracts based on our pre-defined eligibility criteria. We identified 94 articles as potentially eligible; and assessed their full text. Included studies were critically appraised using the Mixed Method Appraisal Tool, version 2011. Fourteen studies were included in the review. We did not identify any meta-analysis or systematic review directly relevant to the question for this systematic review. A narrative approach was used to synthesise the findings of the included studies due to methodological and statistical heterogeneity. Results suggest that certain interventions to enhance the accessibility of homes can have positive health and social effects. Home environments that lack accessibility modifications appropriate to the needs of their users are likely to result in people with physical impairments becoming disabled at home.

## 1. Introduction

The United Nations Convention on the Rights of Persons with Disabilities, Article 9 safeguards the rights of persons with disabilities to live in an accessible physical environment, as well as the right to equal access to information and communications [1]. Among physical environments, there is little doubt that the accessible domestic home is fundamental to enabling independent living for persons with disabilities. Home environments without the basic accessibility components can negatively impact on the daily activities of persons with functional limitations. For instance, those dependent on mobility devices may be confined indoors, or even to very limited spaces within the dwelling; consequently violating their human rights and diminishing their quality of life. It is often assumed that persons with disabilities are a small proportion of the total population, but the World Report on Disability has estimated that more than a billion people, or 15% of the world’s population, have some form of disability [2].

The relationship between ageing and associated functional limitations is becoming increasingly important [3]. The increase in life expectancy over recent decades has resulted in an ageing population especially in high-income countries [4]. More than 20% of the world population is predicted to be aged 60 years or over by the year 2050, with the European region having the highest proportion at an estimated 37% [4]. However, some of the fastest rates of population ageing are now found in low- and middle-income countries [4]. Due to ageing related functional limitations, many older adults face the prospect of living with poor access to their own home environments; threatening their safety and undermining their quality of life. The majority of older adults wish to continue independent living in their own home [5]. However, they are often forced to move into institutional settings due to lack of accessibility to their home environments. Such institutional settings are associated with higher economic costs to both the individual and society in general [6].

According to the International Classification of Functioning (ICF), disability and health, disability is an umbrella term to indicate impairments in body functions and structures, limitations in activities or participation restrictions [7]. Environmental factors (physical, social and attitudinal) can be facilitators or barriers and will determine the level of disability experienced by a person [7]. Disability is not an attribute: it is the outcome of the interaction between bodily impairments and health conditions, and contextual factors (environmental and internal personal factors) [7]. How society is organised, for instance in terms of architectural accessibility, affects whether someone with impairments is “disabled”, or not.

Although the concept of functioning is broad and encompasses impairments, it is often operationalised in terms of whether a person can accomplish Activities of Daily Living (ADL) and Instrumental Activities of Daily Living (IADL) [8]. The term ADL applies to the basic tasks of everyday life, such as bathing, dressing, transferring, toileting and feeding [9,10]. While ADL are more related to personal self-care, IADL refer to a range of activities that are required for independent living in the community, such as preparing meals, housekeeping, taking medications, shopping, managing own finances, travelling and using the telephone [9,10].

It has been estimated that 60% of new houses in the USA are, at some point, likely to be resided in by a person with physical impairment [6]. According to the environmental docility hypothesis, persons with low functional capacity are more likely to be vulnerable concerning environmental demands than are those with higher functional capacity [11]. Therefore, a home without accessibility features creates further strain for persons with functional limitations, increasing their risk of falls and injuries as well as restricting their social participation [12]. Such environments also increase the burden on caregivers and external social services [12,13]. Whereas the built environment and its effects on health and wellbeing have been widely studied [14,15,16], there has been relatively little specific attention to the accessible home environment in the domestic context for persons with functional limitations.

There are various labels that are used for access or accessibility in relation to home environments [17]. For example, Universal Design is defined as the design, construction and adaptation of standard housing that can be used by all people regardless of their age, size or ability [17]. Life Span Housing refers to housing that can accommodate changing capabilities of a person over his/her lifetime, and is also known as Lifetime Homes in the UK and Adaptable Housing in Australia [17]. Enabling technologies for independent living by the elderly has become a new and essential approach, as known as Ambient Assisted Living [18]. For the purpose of this review, we defined the accessible home environment as *one which* *allows a person with functional limitations to get into, out of, and circulate within the home, and to function independently.*

Accessible homes can be purposely built or achieved through modifications, from which various groups of people can benefit: persons with ageing related functional limitations, those with other disabilities, as well as their caregivers and visitors. Furthermore, the importance of an accessible home environment is most likely to increase in coming years and decades because of the increasing prevalence of functional limitations in an ageing population. It is therefore important to evaluate the effects of homes that have accessibility features. This is the objective of the present systematic review. This systematic review is part of a programme of work conducted to support the development of the World Health Organization’s (WHO) Guidelines on Housing.

## 2. Methods

### 2.1. Eligibility Criteria

We addressed the research question using the following structure, which influenced the search strategies used in this review:

**Context:** Domestic home in the community setting regardless of household tenure. Indoor and immediate outside of house, and public spaces and mutual corridors in the case of blocks of flats or buildings. Assisted living facilities, group homes and institutional settings were excluded.
**Participants:** People of all ages who have functional limitations whether physical or cognitive. Frail older adults were included, given that “frail” indicates some forms of impairments. Older adults were excluded if no functional limitations were specified.
**Interventions:** Those implemented in the physical environment of home building that were intended to enhance accessibility: modification of specific furniture and fixture, structural changes, affixed assistive device. Multicomponent interventions and other interventions, e.g., occupational programmes, were included if an accessibility component was incorporated.
**Comparisons:** Groups living in accessible and conventional/unmodified home environments. Comparisons that assessed outcomes before and after an eligible intervention were included.
**Outcomes:** Health or social related changes. Outcomes that were measured jointly regarding home accessibility features and participants’ health/social changes were excluded if they could not be disaggregated.

Searches were conducted in English but there was no language restriction for studies to be eligible. There was no restriction by study type in searching. We planned to limit ourselves to studies with a high level of evidence only if the number of such studies were sufficient for this review. The aim of searching was to identify individual studies and reviews of studies, published as journal articles, technical reports and accessible dissertations. Theoretical papers, commentaries, editorials and abstracts with no full paper were excluded. Book chapters, book reviews and conference proceedings were closely scrutinised as sources for potentially eligible studies.

### 2.2. Data Sources and Search Strategy

Tailored and sensitive search strategies were developed by the expert searcher in liaison with the research team. The search strategy for MEDLINE (Appendix A) was used as the basis for search strategies in the other databases: Cumulative Index of Nursing and Allied Health Literature, Cochrane Database of Systematic Reviews, Cochrane Central Register of Controlled Trials, Database of Abstracts of Reviews of Effects, Health Technology Assessment Database, Embase, OT Seeker, PsycINFO and National Health Service Economic Evaluation Database. Searches were performed from December 2014 to January 2015.

### 2.3. Study Selection

We originally carried out our electronic database searches without any restriction by publication year. However, due to the high number of retrieved records, the WHO Guideline Development Group later set the eligibility to publications to the last 10 years (since 2004), which was more feasible for our review in terms of time frame and budget. Duplicates of records were identified and removed within each database first. After the results from each database had been added to EndNote library, another round of de-duplication was carried out.

Two reviewers independently screened record titles or titles and abstracts based on the pre-defined eligibility criteria, using the EndNote library software programme. Where there was any disagreement or ambiguity, a third reviewer assessed the relevant records and consensus was reached between the three researchers. If it was unclear whether to include or exclude a study on the basis of its abstract, we retrieved its full text. Authors of papers were contacted when more information was required. We checked the reference lists of the included studies, and of systematic reviews that were excluded at the full text screening stage if they concerned home environmental interventions or home interventions on older adult populations.

### 2.4. Data Extraction and Critical Appraisal

After the full text screening process, necessary information was extracted by one reviewer from potentially eligible studies. This included study type, number of participants and their functional limitations, study inclusion and exclusion criteria, interventions and any comparators, outcome measures and results reported. A second reviewer checked data extracted, with any discrepancies resolved by a third reviewer. Potentially eligible studies were then discussed among all the researchers to agree on their relevance to the review.

One of the special characteristics of this review is that such a wide range of study designs were included: studies with no comparison group, correlation studies looking at the association between home accessibility features and outcomes, and mixed-method studies for which results are presented as qualitative themes. Therefore, we used the Mixed Method Appraisal Tool (MMAT), version 2011 to have coherence when assessing the quality of all included studies. The MMAT has been designed to appraise the methodological quality of studies included in complex systematic reviews that incorporate qualitative, quantitative and mixed-method studies [19,20]. The MMAT checklist has two initial screening questions and 19 components corresponding to qualitative research, randomised controlled trial (RCT), non-randomised studies, quantitative descriptive studies and mixed methods studies. It has a scoring metric whereby each study is scored between 1 as the lowest and 4 as the highest quality.

The Evidence Profile was completed using all the information extracted and data from the quality assessment. The summary of findings table was also prepared to identify the effects of interventions for each outcome. All the researchers reviewed and discussed the quality assessment results, the evidence table and summary of findings, easily reaching consensus.

## 3. Results

**Figure 1:**
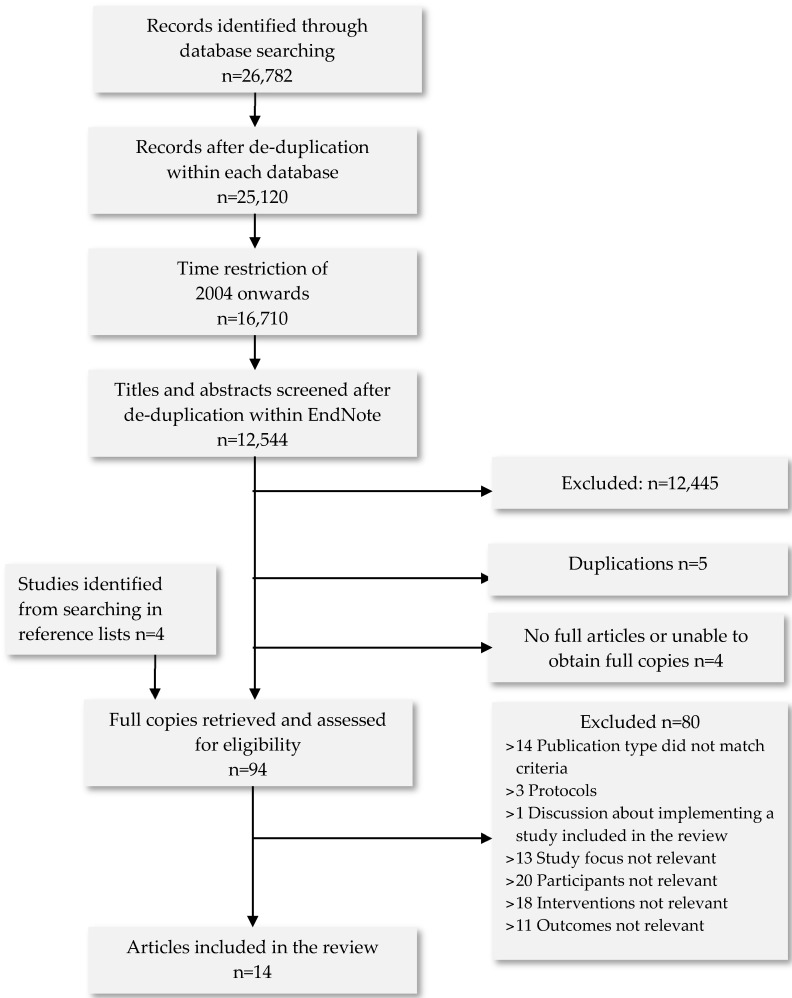
Flow diagram for the identification of eligible studies. (Only one reason is given per excluded study although in many cases reasons for exclusion were more than singular.)

Initially, 26,782 records were identified without any time restriction. After removing records that were published before 2004 and de-duplications, 12,544 records were identified. After titles or titles and abstracts screening, we identified 99 records eligible for the full text screening. Snowballing was also performed and as a result we identified 4 more citations by checking reference lists. Of 103 studies, 5 studies were found to be duplications and 4 with no full articles or unable to obtain full copies. A total of 94 articles were judged to be potentially eligible and therefore we assessed their full text, leading to the exclusion of 80 articles. We did not identify any meta-analysis or systematic review directly relevant to the research question. All researchers agreed on the eligibility of the remaining 14 papers. Figure 1 shows the flow diagram for the identification of studies for this review.

We included all study types in this review as a small number of studies were identified. Table 1 provides a brief presentation of included studies. Full details of characteristics of included studies and their quality assessments are in Appendix B.

### 3.1. Participants

The majority of study participants were elderly population over 70-year old, although inclusion criteria for age groups varied with one study including children [29]. In terms of functional limitations, all study participants had physical impairments except one cross-sectional study that had participants with cognitive impairments [28]. While some studies reported participants with specific functional limitations (such as paraplegia and visual impairments), the majority used diverse terms for and definitions of functional limitations (see Table 2).

### 3.2. Interventions and Home Accessibility Features

Interventions implemented to enhance home accessibility features were home modifications, described as housing adaptations or home safety programmes in some studies. Home modifications were carried out either as a sole intervention [21,22,24,29,30,31,32] or part of a multicomponent programme [25,26,27,34]. Furthermore, the safety component of these, such as hazard reduction, tended to be integrated with the accessibility interventions. Home modifications were mainly focused on architectural changes or fitted devices such as grab bars, targeting mobility issues; a few focused on lighting improvements or adjustments targeting vision. One cohort study had a distinctive intervention which consisted of the installation of a light path near the bed, coupled with tele-assistance: this aimed to reduce falls at night among frail older adults [34]. One randomised trial used a factorial design to evaluate the effect of each intervention, and possible interactions between interventions: home safety programmes; exercise programme; and social visits [23]. Two cross-sectional studies reported the association between accessible home environments and ADL or quality of life [28,33]. Table 3 provides descriptions of accessibility features identified from each included study.

### 3.3. Effects of Interventions on Outcomes

**Figure 2:**
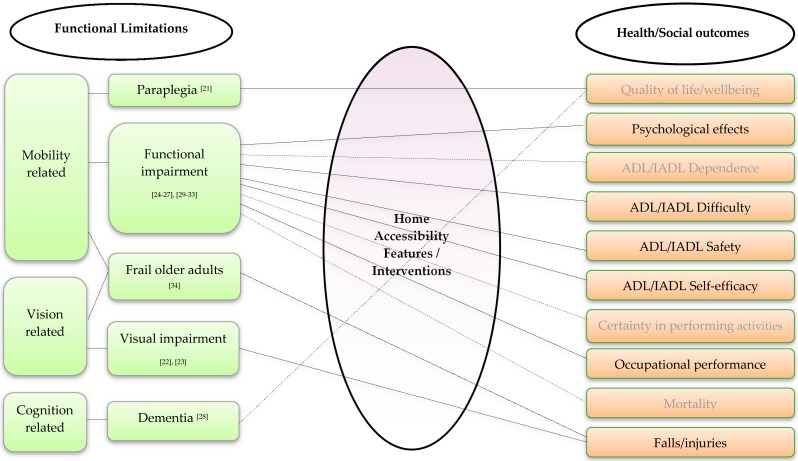
Associations between functional limitations, home accessibility features and outcomes (…. represents no significant or inconsistent associations/effects).

Six different outcomes of home accessibility interventions were identified. The most common outcomes measured were those related to changes in ADL/IADL. Some outcomes were directly related to physical health, such as falls and mortality, and some were related to quality of life and psychological health. Occupational performance was also reported as an outcome of home modifications [32]. All the outcomes were collected via self-report, except mortality that was sourced from the National Death Index [26,27], and fall induced serious injuries which were collected from hospital and general practice records [23]. As will be discussed, Figure 2 schematically illustrates associations between functional limitations categorised into groups (mobility, vision and cognition related) and effects of home accessibility features or interventions on ADL/IADL, occupational performance, falls, mortality, quality of life and psychological health.

### 3.4. Activities of Daily Living

Five studies reported the effects on ADL/IADL related outcomes [22,24,30,31]. In addition, one population-based survey study identified a strong association (odds ratio 3.7, 95% confidence interval, 2.9–4.6) between self-recognised difficulty managing ADL and perceived unmet needs for home accessibility features among people with activity limitations, after adjusting for severity of their limitations [33]. Large decreases in perceived difficulties performing ADL/IADL were identified after home modifications and the multicomponent programme [25,30,31], whereas difficulty with mobility/transfer did not significantly change [25]. Several other aspects in performing ADL/IADL were also reported: safety, dependence, self-efficacy and certainty. Self-efficacy, which was defined as confidence in managing difficulty, was improved in the intervention group after the multicomponent programme among older adults with functional limitations [25]. Increased safety with ADL/IADL was also identified two months after home modifications among adults with functional limitations [30]. In particular, the greatest benefits were in relation to difficulty and safety in bathroom use and entry access [30]. Gitlin 2006a also found that the greatest benefit was in bathing and toileting [25].

On the other hand, two studies found no significant change in dependence with ADL/IADL at 2 months and up to 8–9 months after home modifications [24,30]. However, it was noted that dependence in bathing was significantly decreased between 2–3 months and 8–9 months after home modifications [24]. Furthermore, one randomised trial did not identify a significant improvement overall in self-rated certainty in performing specific activities 6 months after lighting adjustments [22]. Certainty in performing activities of “pour drink“ and “slice bread“ on the working surface of the kitchen were the only ones that improved significantly 6 months after the intervention.

### 3.5. Falls/Injuries and Mortality

Two studies reported on reductions in the likelihood of falls and injuries [23,34]. One randomised trial reported 41% fewer falls by one year follow-up in the home safety programme with a group of older adults with severe visual impairments, compared with those who did not receive this programme [23]. Also, Tchalla 2012 identified a significant reduction in falls at home and post-fall hospitalisations among frail older adults after the use of a light path coupled with tele-assistance [34]. Two studies reported a significantly lower mortality rate at up to 2 years in the intervention group over the control group, after the implementation of the multicomponent programme, which included home modifications as well as training control-oriented strategies to promote healthy behaviours [26,27]. However, there was no statistically significant effect on survival at 3 years post intervention.

### 3.6. Quality of Life

Two randomised trials found a positive effect of interventions on quality of life [21,22]. Ahmed 2013 found that quality of life was significantly enhanced in the intervention group, compared to the control group, 2 months after home modifications among paraplegic wheelchair users [21]. Also, additional lighting adjustments in the living room increased quality of life and wellbeing among adults with low vision [22]. Conversely, a cross-sectional study found no associations between quality of life, and home safety and accessibility factors such as hazards, grab bars and visual cues among adults with dementia [28].

### 3.7. Psychological Effects

Psychological effects of home accessibility interventions were identified. For instance, older adults with functional difficulties reported less fear of falling following multicomponent home intervention [25]. One mixed-method study, which presented findings as themes from the qualitative part of the study, also identified a reduced fear of accidents: 62% of the recipients of minor adaptations (mainly handrails and grab-rails) reported “feeling safer from accidents”, and recipients of major adaptations also expressed the relief of feeling safer [29]. In addition, “ending depression” was identified in the theme of health gains from good quality adaptations for people with physical impairments.

### 3.8. Occupational Performance

A significant increase in self-perceived occupational performance up to 6 months after home modifications among low-income adults with functional limitations was reported [32]. The outcome measurement included self-care (personal care, functional mobility and community management), productivity in work, household and play/school, and leisure (quiet recreation, active recreation and socialisation) [35].

## 4. Discussion

Studies included in this review differ greatly in terms of study designs, participants, interventions and outcomes. Although the majority of the studies’ participants were from the elderly population over 70-year old, the type, definition and level of functional limitations varied. Elements of interventions were remarkably diverse. Despite the fact that mobility related modifications were the most common, some home modifications also included heating or lighting. In addition, it is not clear if the effect of the multicomponent intervention was directly from the accessibility component, and which part of the intervention was more effective. Numerous psychometric instruments were used to measure the same outcomes, such as quality of life and changes in ADL/IADL. This methodological and statistical heterogeneity meant that we adopted a narrative approach to synthesise the findings, rather than performing a meta-analysis.

We found evidence for the positive effect of accessible home environments among people with functional limitations either ageing related or from other causes in this systematic review. Although it contains studies with a low level of quality of evidence, gathering and synthesising the existing evidence will help to guide further research and develop guidelines based on the best evidence available. Overall findings of this review suggest that, in general, people with functional limitations living in accessible home environments have better health, wellbeing and ADL/IADL than those living in conventional or inaccessible home environments. Physical health benefits were identified, such as reductions in falls and injuries. Lower mortality rates were also identified among older adults with functional limitations up to two years after a multicomponent home intervention. Self-perceptions of increased quality of life and general wellbeing were found, along with psychological effects such as reduced fear of falling/accidents and feeling of depression. As fear of falling is known to be a strong risk factor for functional decline and falls [25] this reduction in fear is also an important finding. Furthermore, home modifications decreased difficulties and increased safety and self-efficacy in ADL/IADL outcome measures [25,30,31]. This suggests that people who already have difficulties functioning in everyday life can benefit from home accessibility features, possibly delaying deterioration of their already limited functions.

We did not identify any study reporting the effects of the interventions on dependency on external social care services. Instead, most outcomes were elements in performing ADL/IADL. It seems that longitudinally, improvements in managing ADL/IADL, such as safety, may delay people with impairments being reliant on caregivers or social services. Also, social participation was not directly measured as an outcome in any study. Nevertheless, some psychometric instruments used in the included studies contain rather broad components. For example, occupational performance was reported in one study [32] in terms of performance, and satisfaction with performance in work and leisure. Also, the Client-Clinician Assessment Protocol Part 1, which was used in two studies [30,31], contains a leisure and social activities component, although the remainder is related to ADL, IADL and mobility.

It is noticeable that two studies found no significant change in perceived dependence with ADL/IADL after home modifications [24,30]. This is important because one reason for providing interventions that enhance home accessibility features is to increase the functional independence of people with impairments. However, the participants in both of these studies were aging populations thus their functions may rapidly decline, which means specific home modifications might have an effect for a short period of time only [36]. Furthermore, the primary goal of home modifications for older adults with impairments may be to enable them to live in their own home, rather than increasing their independence per se [30].

Several studies indicated that people with functional limitations received the greatest benefits from interventions in terms of bathroom use, such as bathing, showering and toileting [24,25,30]. This may be because half of ADL tasks focus on the bathroom; and a large number of home adaptations have targeted hygiene facilities [24]. Nonetheless, this is an important finding because it can inform planning for home modifications for people with impairments. Furthermore, Heywood 2004 identified that home modifications that were inadequately implemented due to bad planning or administrative errors, actually had a negative impact on physical and mental health of persons with functional limitations [29]. This indicates that home modification planning should consult with service users as well as health and architectural professionals.

Our search strategy was not restricted to any type of functional limitations but all included studies, except one, were with participants who had physical impairments. During our screening process, it was clear that studies on home environments for people with cognitive impairments were concerned with other environmental matters, such as ‘the creation of safe and secure, simple and well-structured, and familiar environments’ for older adults with dementia [37]. Nevertheless, some of those environmental factors may not necessarily be related to their quality of life: no association was found between patient-perceived quality of life and home accessibility and safety factors among adults with dementia [28]. Instead, having more unmet assistive device/navigation needs and health conditions were associated with lower quality of life [28].

We conducted this systematic review to gather evidence on the effects of the accessible home environment for people with functional limitations, but the findings reach beyond this group. Benefits of accessibility features in the home environments were also apparent for caregivers and family members, who gained positive health impacts, such as greater safety, and prevention of falls and injuries [29]. Furthermore, it is clear that a second person—usually also an older adult—in the household would also use the accessibility features, such as rails or shower [29]. From a population health perspective, this indicates that providing home accessibility interventions may have additional benefits for others; preventing the development of more severe functional limitations, enhancing quality of life and lowering the costs of healthcare. The results of our review are clearly relevant to the ICF framework, given the emphases on the interaction between personal, technological and environmental factors. Furthermore, the results are applicable to the WHO World report on ageing and health, providing evidence that environmental accessibility and safety enable greater functioning in older people [38].

### Study Limitations

There are methodological limitations in the studies included in this review. First, this systematic review included a relatively small number of papers with relatively small sample sizes; making it unfeasible to draw generalised conclusions. Furthermore, the quality of the evidence compiled in this review is quite uneven. Non-randomised studies were included and only four randomised trials of good quality were identified. However, there might be ethical challenges in randomising persons to not receive an intervention or to delay its implementation if there is insufficient uncertainty about the potential benefits of the intervention. It is also important to note that most of the studies included in this review were conducted in the USA and Sweden. While there is no comprehensive national programme and only a few local programmes for home modifications in the USA [17], every local authority in Sweden has to provide home modifications for people with impairments by law [30]. Therefore, the country and systems context in which interventions are evaluated may be quite different, making it impossible to have a control group of people if they have been scheduled for home modifications.

A further limitation is that most of the outcomes in the included studies were subjective self-reports (e.g., ADL/IADL), not objective performance-based measures. However, self-rated function has been found to be useful in clinical assessment as it is predictive of broader health outcomes [39]. In addition, although outcomes are grouped in categories for the reason of convenience, it is important to acknowledge that ADL/IADL related outcomes—such as safety and self-efficacy—are not completely distinct from the psychological effects identified. There are also reliability and validity concerns with some of the psychometric instruments used for ADL/IADL related outcomes, as noted in several papers [22,30,31]. Finally, while the technology used to allow home improvements clearly has some psychologically beneficial effects, related areas have found that the use of assistive technologies, for instance, can present challenging issues concerning user’s self-identity—as being “disabled”—both in terms of how people think about themselves and their own bodily self-image [40,41]. Further exploration of these issues in the context of home improvements may also be worthwhile.

## 5. Conclusions

Home environments that lack accessibility modifications appropriate to the needs of their users are likely to result in people with functional limitations becoming disabled at home. The increasingly aging population means that this is a major concern and also related to the fundamental rights of persons with disabilities. Our systematic review indicates that, in general, interventions to enhance the accessibility of homes can have positive effects. However, currently available research is not robust as a body of evidence and should be considered as providing some support for this finding, albeit with some exceptions. Future research may need to be more specific about type of functional limitations, because different accessibility features may apply to mobility or cognitive impairments for instance. As researchers cannot entirely control the home modification process, it is problematic to conduct controlled studies in the home environment. However, high-quality research is needed, especially longitudinal studies, using standardised outcome measurements, to obtain a stronger evidence base for the benefits of home accessibility interventions. As it is unlikely that improvements to accessibility in the home will be instigated one modification at a time, researchers need to develop more sophisticated designs and analyses in order to partial out the effects of multiple interventions in different types of settings, and health and welfare systems.

## Figures and Tables

**Table 1:** Studies included in the review.

Study	Location	Study Type	Mixed Method Appraisal Tool (MMAT)
Ahmed 2013 [21]	Pakistan	Randomised Controlled Trial (RCT)	**
Brunnström 2004 [22]	Sweden	RCT	***
Campbell 2005 [23]	New Zealand	RCT	****
Fänge 2005 [24]	Sweden	Longitudinal before/after	**
Gitlin 2006a [25]	USA	RCT	****
Gitlin 2006b [26]	USA	RCT	****
Gitlin 2009 [27]	USA	RCT	****
Gitlin 2014 [28]	USA	Cross-sectional	**
Heywood 2004 [29]	UK	Mixed method	** (Quantitative ** Qualitative **)
Petersson 2008 [30]	Sweden	Quasi-experimental pre/post-test	***
Petersson 2009 [31]	Sweden	Quasi-experimental pre/post-test	***
Stark 2004 [32]	USA	Non-randomised before/after	**
Stineman 2007 [33]	USA	Cross-sectional	***
Tchalla 2012 [34]	France	Cohort	**

The MMAT score is presented using descriptors: * as the lowest and **** as the highest quality. This score is the number of criteria met divided by four for qualitative and quantitative studies, and the lowest score of the study components for mixed-method studies.

**Table 2:** Descriptions of functional limitations in studies included.

Types of or Terms Used for Functional Limitations	Definition Provided	Age Group (Years)	Mean Age (Years)
Low vision [22]	Visual acuity ≤0.3 (equal to 6/18)	Adults: no minimum age specified	76
Severe visual impairment [23]	Visual acuity ≤6/24	Older adults ≥75	83.6
Paraplegia [21]	N/A	Adult: no minimum age specified	32.6
Functional limitation [24]	Being considered for housing adaptation	Adults >18	71
Functional impairment [32]	Problems in one or more areas of the Functional Independence Measure motor scale	Older adults: no minimum age specified	70.7
Functional difficulty [25,26,27]	Self-reported difficulties or need for help in at least one in ADL, and at least two in IADL	Older adults ≥70	79 [25,26,27]
Disability [29,30,31,33]	Recipients of housing adaptation [29]	All age groups	71 [29]
	Problems in everyday life and requesting home modifications related to at least one of areas: getting in and out of the home, mobility indoors, self-care in the bathroom [30,31]	Adults ≥40	75.3 [30] 75.1 [31]
	Limitations in kind and amount of activities or work, receipt of any form of insurance or financial support because of disability, limitation in sensation or communication, or use of mobility devices, artificial limb, etc. [33]	Adults >18	Not provided
Frail older [34]	Fried frailty criteria ≥3, and losing functional autonomy as per Functional Autonomy Measure System Profile	Older adults ≥65	83.4
Dementia [28]	Not provided	Adults: no minimum age specified	82

**Table 3:** Descriptions of accessibility features in each study included.

Intervention	Accessibility Features	Related Function
Home modification as a sole intervention	Targeting hygiene facilities (installation of grab bars in the bathtub or shower, replacing the bathtub with a shower), entrances including balcony and patio, stairways and doors (automatic door openers). A few adaptations targeting floor surfaces in bathrooms.	Mobility [24,30,31]
	Wheelchair accessible doors, ramps, rails, tub seat in bathrooms, non-slip surface	Mobility [21]
	Handrails, grab bars, ramps, hand-held shower, raised toilet, roll-in shower, widen door, relocating laundry facilities to ground floor, bed rail, designated parking area on street Lever handles on doors Additional lighting Safety features (deadbolts, smoke detectors) and adaptive equipment (reachers, tub benches) included	Mobility & vision [32]
	Lighting adjustments in the kitchen, bathroom, hall and living room	Vision [22]
	Reducing glare, improving lighting Painting the edge of steps Installation of grab bars, stair rails Removing or changing loose floor mats, removing clutter	Vision & mobility [23]
	Minor adaptations: handrails, grab-rails Major adaptations: stair-lifts, bathroom conversions providing level-access shower, extensions to provide ground-floor bedroom, bathroom or both, stair-and through-floor lifts, installations of downstairs toilets, door widening, ramps, kitchen alteration Heating included	Mobility [29]
Multi-component interventions	Installation of grab bars, rails, raised toilet seats Occupational therapy sessions (training of problem solving strategies, energy conservation, safe performance, fall recovery technique) and physiotherapy sessions	Mobility [25,26,27]
	Light path installed near the bed with tele-assistance	Vision [34]
N/A (Cross-sectional studies)	Home Environmental Assessment Protocol: hazards (access to dangerous objects), adaptation (grab bars, visual cues)	Cognition [28]
	Environmental accessibility barriers: wide doorways, ramps, railings, automatic doors, elevators, bathroom, kitchen or other modification	Mobility [33]

## Appendix A. Search Strategy for Ovid MEDLINE

In-Process & Other Non-Indexed Citations and Ovid MEDLINE(R) <1946 to Present>, Search date: 22 December 2014, Records identified: 6829 (5635 after de-duplication)

1. exp Disabled Persons/ (48958)
2. exp housing/ (26214)
3. 1 and 2 (426)
4. (home or homes or house or houses or housing or residen$ or built environment or living environment).ti. (111898)
5. 1 and 4 (1382)
6. architectural accessibility/or “Facility Design and Construction”/or residence characteristics/or environment design/ (34524)
7. 1 and 6 (1156)
8. ((home or homes or house$ or housing or residen$) adj2 (adapt$ or modif$ or access$ or usability)).ti,ab. (2117)
9. (smart home$ or smart home technolog$).ti,ab. (193)
10. (assistive technolog$ and (home or homes or house or houses or housing or residence$ or built environment$ or living situation)).ti,ab. (163)
11. environmental barrier$.ti,ab. (430)
12. universal design.ti,ab. (148)
13. (disability or disabled or handicap$).ti,ab. (129312)
14. 2 and 13 (410)
15. ((disability or disabled or handicap$ or frail$) adj2 (home or homes or house or houses or housing or residen$ or environment)).ti,ab. (592)
16. (home environment$ adj2 intervention$).ti,ab. (15)
17. (environment$ intervention$ adj2 home$).ti,ab. (26)
18. person environment$ fit.ti,ab. (139)
19. person-environment$ fit.ti,ab. (139)
20. person environment$-fit.ti,ab. (139)
21. person-environment$-fit.ti,ab. (139)
22. (home or homes or house or houses or housing or residen$ or built environment or living environment).ti,ab. (378471)
23. (functional$ adj (handicap$ or impair$ or limit$ or decline$ or deficit$ or disable$ or disability)).ti,ab. (28806)
24. (cognitive$ adj (handicap$ or impair$ or limit$ or decline$ or deficit$ or disable$ or disability)).ti,ab. (57923)
25. (mental$ adj (handicap$ or impair$ or limit$ or decline$ or deficit$ or disable$ or disability)).ti,ab. (5162)
26. (physical$ adj (handicap$ or impair$ or limit$ or decline$ or deficit$ or disable$ or disability)).ti,ab. (8385)
27. (motor adj (handicap$ or impair$ or limit$ or decline$ or deficit$ or disable$ or disability)).ti,ab. (11157)
28. (hearing adj (reduc$ or loss or handicap$ or impair$ or limit$ or decline$ or deficit$ or disable$ or disability)).ti,ab. (39532)
29. ((vision or visual or sight) adj (reduc$ or loss or handicap$ or impair$ or limit$ or decline$ or deficit$ or disable$ or disability)).ti,ab. (21276)
30. (blind or deaf or frail$).ti,ab. (173515)
31. wheelchair user$.ti,ab. (856)
32. amputee$.ti,ab. (4124)
33. 23 or 24 or 25 or 26 or 27 or 28 or 29 or 30 or 31 or 32 (338691)
34. 2 and 33 (331)
35. (((functional$ adj (handicap$ or impair$ or limit$ or decline$ or deficit$ or disable$ or disability)) or (cognitive$ adj (handicap$ or impair$ or limit$ or decline$ or deficit$ or disable$ or disability)) or (mental$ adj (handicap$ or impair$ or limit$ or decline$ or deficit$ or disable$ or disability)) or (physical$ adj (handicap$ or impair$ or limit$ or decline$ or deficit$ or disable$ or disability)) or (motor adj (handicap$ or impair$ or limit$ or decline$ or deficit$ or disable$ or disability)) or (hearing adj (reduc$ or loss or handicap$ or impair$ or limit$ or decline$ or deficit$ or disable$ or disability)) or ((vision or visual or sight) adj (reduc$ or loss or handicap$ or impair$ or limit$ or decline$ or deficit$ or disable$ or disability)) or (blind or deaf) or wheelchair user$ or amputee$) adj (home or homes or house or houses or housing or residen$ or built environment)).ti,ab. (170)
36. wheelchairs/ (3833)
37. 2 and 36 (27)
38. 22 and 36 (246)
39. communication aids for disabled/ (2187)
40. 2 and 39 (6)
41. 22 and 39 (82)
42. (mobility adj (impair$ or device$ or aid$)).ti,ab. (934)
43. 2 and 42 (6)
44. 22 and 42 (171)
45. 3 or 5 or 7 or 8 or 9 or 10 or 11 or 12 or 14 or 15 or 16 or 17 or 18 or 19 or 20 or 21 or 34 or 35 or 37 or 38 or 40 or 41 or 43 or 44 (6931)
46. (rat or rats or mouse or mice or poultry or pig or pigs or cat or cats or sheep or cow or cows).ti. (1370530)
47. 45 not 46 (6829)

## Appendix B. Characteristics of Included Studies

**Table:**

**Study: Ahmed 2013**		**Title: Effectiveness of Home Modification on Quality of Life on Wheelchair User Paraplegic Population**			
**Authors: Junaid Ahmed, Syed Shakil-ur-Rehman, Fozia Sibtain**					
Study type	Setting	Inclusion criteria	Definition of specific functional limitation	Exclusion criteria	Recruitment procedures
RCT	District Kohat & Hangu in Pakistan January–December 2012	Paraplegic adult wheelchair users	N/A	Insufficient information provided.	Insufficient information provided.
Samples	Interventions		Outcome measures	Results	Quality (MMAT) & Limitations
*N* = 40 *n* = 20 home modification (mean age: 33.66 years) *n* = 20 control (mean age: 31.57 years)	The intervention group received home modifications: wheelchair accessible doors, ramps, rails, tub seat in bathrooms, & non-slip surface.		Modified LiSAT questionnaire (6 point scale): life as a whole, vocational situation, financial situation, leisure situation, contact with friends and relatives, ability to manage self-care, family life. Before and 2 months after the intervention.	SPSS v 20 and paired *t*-test used at significance level 5%. Quality of life significantly enhanced in the experimental group, compared to the control group: LiSAT score 33.32 (*p* = 0.001) vs. 22.85 (*p* = 0.154). No SD or CI specified.	MMAT ** (Insufficient information provided on randomisation, sequence generation or allocation concealment.) Small sample size unlikely represents the target population.
**Study: Brunnström 2004**		**Title: Quality of light and quality of life—The Effect of Lighting Adaptation among People with Low Vision**			
**Authors: Gunilla Brunnström, Stefan Sorensen, Karin Alsterstad, John Sjostrand**					
Study type	Setting	Inclusion criteria	Definition of specific functional limitation	Exclusion criteria	Recruitment procedures
RCT	Goteborg, Sweden	Adults with low vision	Visual acuity ≤0.3 (6/18)	Insufficient information provided.	Participants were consecutively recruited from those receiving lighting adaptation by the Low Vision Clinic at Sahlgren University Hospital.
Samples	Interventions		Outcome measures	Results	Quality (MMAT) & Limitations
*N* = 56 recruited: Nine dropped out before randomisation and one before the first stage. *N* = 46 (mean age 76 years, range 20–90 years) *n* = 24 intervention *n* = 22 comparison Macular degeneration dry form (*n* = 12), macular degeneration wet form (*n* = 16), retinitis pigmentosa (*n* = 2), glaucoma: (*n* = 5), and other diagnoses (*n* = 11)	The intervention group received lighting adjustment in the kitchen, bathroom and hall according to a pre-determined measurement protocol. They received an additional lighting adjustment in the living room. Controls received lighting adjustment in the kitchen, bathroom and hall. They did not receive the additional lighting adjustment.		Perceived certainty in performing activities (7 points): pouring a drink, slicing bread, regulating the cooker, findings things finding cupboards, on the table, and plate Perceived certainty in performing activities (yes/no): preparing food, washing up, laying the table, looking in the mirror (bathroom), seeing if clothes are dirty, matching items of clothing Reading the newspaper Psychological and general well-being (PGWB) scale: seven points Participants were interviewed before and 6 months after the intervention.	Seven point scale daily activities tested using Wilcoxon signed ranks test, and OR and 95% CI used for yes/no activities. Overall, no significant change in perceived activity performance in the kitchen and bathroom in both groups. Only the activities on the working surface in the kitchen improved significantly: “pour drink” Median difference Md 1.5 to 3.5, *p* = 0.03, “slice bread” Md 3.0 to 6.0, *p* = 0.04. Quality of life tested using Wilcoxon signed ranks test at significance level 5%. Comparison group had no change in quality of life and well-being, whereas the intervention group showed a significant improvement for all items (range *p* = 0.01–0.04). No CI specified.	MMAT *** Small sample size unlikely represent the target population. Differences between groups for demographic characteristics not specified. Samples were heterogeneous in terms of diagnosis. Approximately half of the participants reported that their perceived eyesight had worsened during the actual study period. It might have affected their activity function. Validity and reliability issues of psychometrics used (ADL and quality of life).
**Study: Campbell 2005**		**Title: Randomised Controlled Trial of Prevention of Falls in People Aged ≥75 with Severe Visual Impairment: The VIP Trial**			
**Authors: A John Campbell, M Clare Robertson, Steven J La Grow, Ngaire M Kerse, Gordon F Sanderson, Robert J Jacobs, Dianne M Sharp, Leigh A Hale**					
Study type	Setting	Inclusion criteria	Definition of specific functional limitation	Exclusion criteria	Recruitment procedures
RCT 2 × 2 factorial design	Dunedin & Auckland, New Zealand Recruitment period: over 12 months from October 2012	Older adults ≥ 75 with severe visual impairment	Visual acuity ≤6/24	Those who could not walk around their own residence Those who were receiving physiotherapy Those who could not understand the trial requirement	Participants were recruited through records from the blind register, low vision clinics and hospitals.
Samples	Interventions		Outcome measures	Results	Quality (MMAT) & Limitations
*N* = 391 *n* = 100 home safety programme only (mean age 83.1 years) *n* = 97 exercise programme (mean age 83.4 years) *n* = 98 both home modification & exercise (mean age 83.8 years) *n* = 96 social visits (mean age 84.0 years)	Home safety programme: Occupational Therapist visited home, carried out home safety assessment, made recommendations to implement and facilitated payment for home modification. 90% of participants (152/169) reported complying partially or completely with one or more of the recommendations: removing or changing loose floor mats, painting the edge of steps, reducing glare, installing grab bars and stair rails, removing clutter, and improving lighting. Exercise programme included modified Ontago exercise for a year with vitamin D supplementation.Social visits included two 60 min lasting home visits.		Number of self-reported falls, and injuries resulting from falls Economic evaluation One year follow-up	Negative binomial regression models used. 41% fewer falls in the home safety programme only group compare with those who did not receive this programme (incident rate ratio 0.59, 95% CI 0.42 to 0.83); exercise programme (incident rate ratio 1.15, CI 0.82 to 1.61). No significant difference in the reduction of falls at home compared to outside home environment. Neither intervention was effective in decreasing fall related injuries. The home safety programme costed $NZ 650 (£234, 344 euro, $US 432 at 2004 prices) per fall prevented.	MMAT **** The duration of visual impairment varied significantly. Participants’ abilities were not taken into account for participating in an exercise programme.
**Study: Fänge 2005**		**Title: Changes in ADL Dependence and Aspects of Usability Following Housing Adaptation—A Longitudinal Perspective**			
**Authors: Agneta Fange, Susanne Iwarsson**					
Study type	Setting	Inclusion criteria	Definition of specific functional limitation	Exclusion criteria	Recruitment procedures
Longitudinal, before and after	Medium sized municipality in southern Sweden with urban and rural areas.	Adults >18 with functional limitations	Those who were being considered for housing adaptation grants.	Terminally ill clients Clients who spent most of the in a bed or chair Clients with communication problem	Clients were consecutively enrolled over 18 months, who applied for housing adaptation grants.
Samples	Interventions		Outcome measures	Results	Quality (MMAT) & Limitations
*N* = 131 (88 female, mean age 71 years) 2–3 months follow-up: *N* = 104 8–9 months follow-up: *N* = 98	Housing adaptation grants administered. The majority of the adaptations targeting hygiene facilities (installation of grab bars at the bathtub or shower, replacing the bathtub with a shower), entrances including balcony and patio, and stairways and doors. A few adaptations targeting floor surfaces in bathrooms.		ADL staircase, Revised version that comprises 5 personal ADL and 4 IADL, 3 graded scale (independent, partly dependent, dependent) Usability in My Home Instrument: environmental impact on performance of ADL/IADL, 23 items in total with 16 of 7-point scale and 7 of open-ended questions Before (T1), 2–3 months after (T2), 8–9 months after the intervention (T3).	ADL ranks and changes in overall as well as in each ADL item were analysed by means of the Sign test at significance level 5%. No significant change in overall ADL dependence at any time point relative to baseline, whereas dependence in bathing decreased between T2 and T3 (*p* = 0.0020). Usability: No significant change in activity aspects between T1 and T3, although great improvement between T1and T2 (*p* = 0.045). Significant improvement in personal and social aspects between T2 and T3 (*p* = 0.008), although no changes earlier.	MMAT ** Small sample size may explain the lack of significant changes over time. No comparison group. Other interventions may have been implemented on the participants: mobility devices were prescribed from other interventions during the home modification process.
**Study: Gitlin 2006a**		**Title: A Randomized Trial of a Multicomponent Home Intervention to Reduce Functional Difficulties in Older Adults**			
**Authors: Laura N. Gitlin, Laraine Winter, Marie P. Dennis, Mary Corcoran, Sandy Schinfeld, Walter W. Hauck**					
Study type	Setting	Inclusion criteria	Definition of specific functional limitation	Exclusion criteria	Recruitment procedures
RCT	Urban, United States Participants were recruited 2000–2003	Older adults ≥70 who reported difficulty with one or more activities of daily living and were ambulatory	Self-reported difficulties or need for help: one or more in ADLs, and two or more in IADLs	MMSE ≤23 Non-English speaking people Those who were receiving home care	Participants were recruited from an area agency on aging and advertisements through media and posters.
Samples	Interventions		Outcome measures	Results	Quality (MMAT) & Limitations
*N* = 319 (mean age 79) *n* = 160 intervention (mean age 79.5) *n* = 159 control (mean age 78.5) Follow-up 1(6 months): *N* = 300 (94%) Follow-up 2 (12 months): *N* = 285 (89%)	The intervention group received home occupational (four 90 min visits and one 20 min telephone contact) and Physical Therapy sessions (one 90 min) during the first 6 months. OT/PT sessions included home modifications (e.g., grab bars, rails, raised toilet seats) and training; instruction in problem solving strategies, energy conservation, safe performance, fall recovery technique, and balance and muscle strength training. Control: no treatment Home modifications were paid for through grant funds.		ADL, mobility/transferring, and IADL: 5 point scale, perceived difficulty Tinetti et al.’s Falls Efficacy Scale, and three items from Powell et al.’s Activities-specific Balance Confidence Scale: 10-point scale, perceived fear of falling Self-efficacy: confidence in managing ADL, IADL and mobility, 5 point scale Secondary: observed home hazards, use of adaptive strategies Before and at 6 months and 12 months.	At 6 months, the intervention group reported less difficulty than controls with ADL (*p* = 0.03, 95% CI = −0.24 to −0.01) and IADL (*p* = 0.04, 95% CI = −0.28–0.00). The biggest benefits were in bathing (*p* = 0.02, 95% CI = −0.52 to −0.06) and toileting (*p* = 0.049, 95% CI = −0.35–0.00). No significant change in mobility/transfer difficulty. The intervention group had greater self efficacy (*p* = 0.03, 95% CI = 0.02–0.27), less fear of falling (*p* = 0.001, 95% CI = 0.26–0.96), and greater use of adaptive strategies (*p* = 0.009, 95% CI = 0.03–0.22). 12-months effects similar to those at 6 months.	MMAT **** The study participants were voluntary: they might have been more motivated. As it was the multicomponent intervention, it is unclear if one intervention was more effective than others. Use of a no-treatment control group: attention from health professionals may account for beneficial effects.
**Study: Gitlin 2006b**		**Title: Effect of an in-Home Occupational and Physical Therapy Intervention on Reducing Mortality in Functionally Vulnerable Older People: Preliminary Findings**			
**Authors: Laura N. Gitlin, Walter W. Hauck Laraine Winter, Marie P. Dennis, Richard Schulz**					
Study type	Setting	Inclusion criteria	Definition of specific functional limitation	Exclusion criteria	Recruitment procedures
14 months follow-up of RCT (Gitlin 2006a)	Urban, Philadelphia, United States Participants were recruited 2000–2003	Older adults ≥70 with functional difficulties and were cognitively intact	Functional vulnerability: needing help with two IADLs, having difficulty performing one ADL, or experiencing one or more falls within 1 year before study entry	MMSE ≤23 Non-English speaking Who were receiving home care	Participants were recruited from local social service agencies, an area agency on aging, and media announcements.
Samples	Interventions		Outcome measures	Results	Quality (MMAT) & Limitations
*N* = 319 (mean age ± standard deviation 79 ± 5.9) Female 62%, living alone 62% *n* = 160 intervention (mean age 79.5) *n* = 159 control (mean age 78.5)	The intervention group received home occupational (four 90 min visits and one 20 min telephone contact) and physical therapy sessions (one 90 min) during the first 6 months. OT/PT sessions included home modifications (e.g., grab bars, rails, raised toilet seats) and training; instruction in problem solving strategies, energy conservation, safe performance, fall recovery technique, and balance and muscle strength training. Control: no treatment Home modifications were paid for through grant funds.		Health and physical function: health conditions, days hospitalised 6 months before study entry, self-rated health, formal services, medications, emergency visits, days in rehabilitation, difficulty in ADL, IADL and mobility/transfer Mortality over 14 months Control-oriented strategy use	The intervention group had a significantly lower mortality rate than controls: 1% vs. 10% (*p* = 0.003, 95% CI 2.4–15.04). No one from the intervention group with previous days hospitalised (*n* = 31) died, whereas 21% of control group counterparts did (*n* = 35; *p* = 0.001). Mortality risk was lower for intervention participants with low strategy use at baseline (*p* = 0.007).	MMAT **** Cause of death generally not known. Health professionals might have detected medical problems and recommended treatment for intervention subjects. Exploratory analysis, this was not planned. Subjective self-reports of functional difficulties were used. The number of deaths that occurred in the study period was modest (*n* = 14).
**Study: Gitlin 2014**		**Title: Correlates of Quality of Life for Individuals with Dementia Living at Home: The Role of Home Environment, Caregiver, and Patient-Related Characteristics**			
**Authors: Laura N. Gitlin, Nancy Hodgson, Catherine Verrier Piersol, Edward Hess, Walter W. Hauck**					
Study type	Setting	Inclusion criteria	Definition of specific functional limitation	Exclusion criteria	Recruitment procedures
Cross-sectional	Urban, East Coast region, United States Participants were enrolled June 2009–October 2010.	Adults with dementia Caregivers ≥21 years; lived with/in close proximity to patients; English speaking; Provided care for 5 months or more	Insufficient information provided	For patients MMSE <10 Those who were bed-bound or unresponsive Those who could not speak English	Participants were recruited through media advertisements and mailings by aging and faith-based organisations, targeting caregivers.
Samples	Data collection		Outcome measures	Results	Quality (MMAT) & Limitations
*N* = 88 dyads (97%) completed two home assessments and are included in the analysis *n* = 88 patients (mean age 82 years, range 56–97) *n* = 88 caregivers (mean age 65.8, range 38–89)	All participants received a 45-min telephone interview, 90-min first home visit with MMSE administration, and a second visit within 2 weeks of completion of interviews.		Quality of Life in Alzheimer Disease: 4 point scale Home Environmental Assessment Protocol: home hazards (access to dangerous objects), adaptations (grab bars, visual cues), measured via observation or interviews, two indices represent the total number of hazards and adaptation Unmet home environmental needs by asking two yes/no questions to caregivers Patient-related factors: health conditions, behavioural frequency, fall risk, pain & sleep quality Caregiver-based factors: mood, positive caregiving, & communication	Linear regression model used, two sided, at significance level 5%. Home environmental factors were not associated with perceived quality of life: adaptation (Regression Coefficient B = −0.284, 95% CI −0.647 to 0.079, *t* = −1.558, *p* = 0.123), hazards (B = 0.002, 95% CI −0.292 to 0.296, *t* = 0.016, *p* = 0.987). Environmental factors were not associated with caregiver-perceived quality of life of patients. Having more unmet assistive device/navigation needs (B = −2.314, 95% CI −4.370 to -0.258, *t* = −2.240, *p* = 0.028) and health conditions (B = −0.707, 95% CI −1.161 to −0.253, *t* = −3.101, *p* = 0.003) were associated with patient-perceived lower quality of life in separate regressions.	MMAT ** Small sample size and cross-sectional design. Not all modifiable and relevant factors were included in this study.
**Study: Heywood 2004**		**Title: The Health Outcomes of Housing Adaptations**			
**Authors: Frances Heywood**					
Study type	Setting	Inclusion criteria	Definition of specific functional limitation	Exclusion criteria	Recruitment procedures
Mixed method: interviews and questionnaires	England and Wales in the UK Field work 1999–2000	Recipients of housing adaptation	No definition or description of disability types provided, although the term of “disabled people” are used in this article.	Insufficient information provided.	Participants were recruited through social services or housing authorities records.
Samples	Data collection		Analysis	Results	Quality (MMAT) & Limitations
*N* = 104 interviews (84 face-to-face and 20 telephone) *N* = 162 questionnaires (mean age 71 years, women 115) NB: There is a primary report (Heywood 2001) of this research study with more information on samples and interventions. This article focuses on health related findings.	Combination of structured and semi-structured interviews, also asked to give a score out of 10 for the effect of adaptation. The pairs of interviewers agreed a score themselves. 104 interviews with recipients of major home adaptations and 162 postal questionnaires by recipients of minor adaptations in six out of seven areas. Minor adaptations: quickly and easily fitted fixed alteration costing less then £500, e.g., hand-rails, grab-rails. Major adaptations: stair-lifts, bathroom conversions (usually providing a level-access shower, extensions to provide ground-floor bedroom, bathroom or both, stair- and through-floor lifts, the installation of a downstairs toilet, door widening, ramps, kitchen alterations. Home modifications included heating.		SPSS database used for establishment of core frequencies and links. Then, an adapted version of the NCSR framework methodology was used, involving repeat reading of interview transcripts to identify themes. Searches from the themes on words or groups of words were carried out to check frequency.	Key themes: Health impacts on disabled people before housing adaptation or after inadequate adaptation: pain, accident, exacerbated illness, feeling of depression Health impacts on caregivers & other family members: injuries, falls Health gains from good quality adaptations for disabled people: relief of pain, preventing accidents & reducing fear of accidents, ending depression Health benefits to other household members Inter-active effects	MMAT overall **: Qualitative **, Quantitative **, Mixed Method ** Low response rate for questionnaires: 60%. Questions were sent to participants in advance for interviews.
**Study: Petersson 2008**		**Title: Impact of Home Modification Services on Ability in Everyday Life for People Ageing with Disabilities**			
**Authors: Ingela Petersson, Margareta Lilja, Joy Hammel, Anders Kottorp**					
Study type	Setting	Inclusion criteria	Definition of specific functional limitation	Exclusion criteria	Recruitment procedures
Quasi-experimental pre-post test Part of a larger ongoing longitudinal research project	A large city in Sweden Data were collected 2002–2005	Adults ≥40 with disabilities	Problems in everyday life and requesting home modifications related to at least one of the followings 3 areas: Getting in & out of the home Mobility indoors Self-care in the bathroom	MMSE <19 CES-D depression ≥24 Those who could not communicate in Swedish	The Home Modification (AHM) identified potential participants.
Samples	Interventions		Outcome measures	Results	Quality (MMAT) & Limitations
Baseline: *N* = 114, *n* = 73 intervention, *n* = 41 comparison group Follow-up: *N* = 105 (mean age 75.3) *n* = 73 intervention, (mean age 75.7 years) *n* = 41 comparison (mean age 74.6 years)	Those who have been scheduled for home modifications within 4 weeks were allocated in the intervention group, and received home modifications as scheduled. Common home modifications included shower, ramps and automatic door openers. Those who were waiting for their application to be investigated by the AHM were allocated in the comparison group. They did not receive home modifications during the time of the study. All cost were covered for modifications by the local authorities.		Client–Clinician Assessment Protocol (C-CAP) Part I: self-rated independence (4-point scale), difficulty (5-point scale) and safety (3-point scale) in ADL, IADL, mobility & leisure Before and 2 months after the intervention	Paired sample *t*-tests used with a level of significance level at *p* < 0.05. Intervention group had a significant increase of safety (*t* = −3.820 p = 0.001 effect size *d* = 0.40) and decrease of difficulty (*t* = −3.353 *p* = 0.001 *d* = 0.32) in ADL. No significant change in self-rated functional independence in the intervention group (*t* = −0.630 *p* = 0.531). Specifically, decreased difficulties and increased safety in bathroom use, and getting in and out of house. Self-rated safety in taking medication was significantly decreased in the intervention group. No significant change in abilities in the comparison group.	MMAT *** Small sample size and urban living samples that applied for home modifications might not be generally representative. Psychometric limitations in the C-CAP Part I: validity issue. Unclear whether self-rated improvements in everyday life were directly from home modifications, or were related to other factors, e.g., technical devices.
**Study: Petersson 2009**		**Title: Longitudinal Changes in Everyday Life after Home Modifications for pEople Aging with Disabilities**			
**Authors: Ingela Petersson, Anders Kottorp, Jakob Bergstrom, Margareta Lilja**					
Study type	Setting	Inclusion criteria	Definition of specific functional limitation	Exclusion criteria	Recruitment procedures
Quasi-experimental pre-post test	A large city in Sweden Data were collected 2002–2005	Adults ≥40 with disabilities	Problems in everyday life and requesting home modifications related to at least one of the followings 3 areas: Getting in & out of the home Mobility indoors Self-care in the bathroom	MMSE <19 CES-D depression ≥24 Those who could not communicate in Swedish	The local Agency for Home Modification (AHM) identified potential participants. Those who have been scheduled for home modifications within 4 weeks: intervention group Those who were waiting for their application to be investigated by the AHM: comparison
Samples	Interventions		Outcome measures	Results	Quality (MMAT) & Limitations
Baseline: *N* = 103 (mean age 75.1 years), *n* = 74 intervention (mean age 75.19 years), *n* = 29 comparison (mean age 74.5 years) Follow-up 1: *N* = 94, *n* = 69 intervention, *n* = 25 comparisonFollow-up 2: *N* = 84, *n* = 64 intervention, *n* = 20 comparison	Intervention group received home modifications as scheduled. Common home modifications included shower, ramps and automatic door openers. Comparison group did not receive home modifications during the time of the study. In Sweden, the local authorities are obliged to provide home modifications in the form of a grant to people with disabilities. All cost are covered for modifications		Self-rated Difficulty scale of the Client–Clinician Assessment Protocol (C-CAP) Part I: only difficulty part used, 5-point scale Before, 2 months after and 6 months after home modifications	Random coefficient models used. Intervention group had less difficulty up to 6 months than the comparison group: intervention vs. comparison mean difference Logits = 0.450 SE = 0.156 *p* = 0.023 95% CI 0.082 to 0.819 Small to moderate effect size for home modifications for the intervention group at both follow-up: follow-up 1 (Mean = 0.35 SE = 0.15 *d* = 0.34) & follow-up 2 (Mean = 0.37, SE = 0.16, *d* = 0.0.32) No effect in the comparison group. One confounding factor, waiting time for home modifications had an additional impact on experienced difficulties in ADL	MMAT *** Small sample size, large dropout in the comparison group, and urban living samples might not be generally representative. Psychometric limitations in the C-CAP Part I. Difficulty of measuring whether self-rated improvements in everyday life were directly as a result from home modifications, or were related to other factors, e.g., technical devices.
**Study: Stark 2004**		**Title: Removing Environmental Barriers in the Homes of Older Adults with Disabilities Improves Occupational Performance**			
**Authors: Susan Stark**					
Study type	Setting	Inclusion criteria	Definition of specific functional limitation	Exclusion criteria	Recruitment procedures
Non-randomised pre-post	Urban area in United States 1999–2000	Low income older adults with functional impairments and indicated a need for environmental modifications	Problems in one or more areas of the Functional Independence Measure (FIM) motor scale	Cognitive subscale of the FIM ≤ 25	Participants were identified by a not-for-profit agency that provides free or low cost architectural (accessibility) modifications in partnership with occupational therapists.
Samples	Interventions		Outcome measures	Results	Quality (MMAT) & Limitations
*N* = 29 (age range 57–82 years, mean age 70.69 years) 16 participants were retained in the study: *n* = 12 African Americans *n* = 12 women	Participants received occupational therapy home modification programme, an average of 2.5 home modifications per person, ranging from 1–7. Most common modifications were the installation of handrails, grab bars and ramps. Less common modifications included bedrails, widening doors, relocating laundry facilities from the basement to the living floor, and additional lights. Interventions were limited to compensatory strategies only. No other remedial intervention. If participants were able to pay for home modifications, they did so. If not, the agency provided it at no cost.		Canadian Occupational Performance Measure (COPM) via semi-structured interviews and structured scoring method (10-point scale). Participants were asked about importance, performance and satisfaction in self-care (personal care, functional mobility and community management), productivity in work, household and play/school, and leisure (quiet recreation, active recreation and socialisation) Baseline data collection: Severity of disability by the FIM, COPM, Environmental Functional Independence Measure (Enviro-FIM) assessed by interviews and observations. Before, 3 months after and 6 months after home modifications.	Paired *t* tests used to examine the differences between pre and post intervention. Participants’ self-perceived occupational performance (*t* = −8.23 *p* = 0.0001) and satisfaction with performance (*t* = −9.54 *p* = 0.0001) increased significantly at 6 months.	MMAT ** Small sample size and limited follow-up, longitudinal studies may be required regarding health status changes over time. No control group. Participants were mainly African American: not representative of the general population of older adults with disabilities. Lengthy time lapse from enrolments to completion of modifications may have allowed changes in physical status.
**Study: Stineman 2007**		**Title: Population-Based Study of Home Accessibility Features and the Activities of Daily Living: Clinical and Policy Implications**			
**Authors: Margaret G. Stineman, Richard N. Ross, Greg Maislin, David Gray**					
Study type	Setting	Inclusion criteria	Definition of specific functional limitation	Exclusion criteria	Recruitment procedures
Cross-sectional (survey)	United States Phase I: August 1994–1997 Phase II: 206–722 days later, limited to persons with disabilities	Adults>18 with disabilities, non-institutionalised, answered all survey questions themselves, and described at least one physical limitation (Phase II of the National Health Interview Survey (NHIS) supplements on Disability (NHIS-D))	Limitations in kind and amount of activities or work, receipt of any form of insurance or financial support because of disability, limitations in sensation or communication, or use of mobility devices, artificial limb, etc.	Those who were institutionalised and ≤18	Data from phase I and II of NHIS-D: Phase I was representative of the US non-institutionalised civilian population > 18 years. Phase II was limited to persons with disabilities. Phase II data was used to address person-environmental interactions.
Samples	Data collection		Outcome measures	Results	Quality (MMAT) & Limitations
*N* = 25,805 in Phase II	80% (*n* = 20,644) randomly assigned to a model building sample, and 20% (*n* = 5161) to a validation data. 7922 (85%) in the model building data met all the criteria, and had all variables necessary for primary analysis. This made up the samples on which the effects of environmental barriers were modelled: 1952 respondents in the validation data set who met the same criteria.		Outcome measure Self-reported difficulty or inability in ADLs Primary predictors: Self-perceived environmental barriers: wide doorways, ramps into the home, railings inside the home, automatic doors, elevators, bathroom, kitchen or other modification Physical limitations: lower boy use, hand use and reaching Assistive technology: limited to mobility aids Socioeconomic variable	There were 12,743 people with physical impairments, 10.3% of whom perceived an unmet need for at least o 1 home accessibility feature. After adjusting for severity of physical limitation and socioeconomic differences, the odds of an ADL difficulty were 3.7 times larger (95% CI 2.9–4.6) among participants who perceived an unmet need for accessibility features.	MMAT *** It was restricted to physical limitations only and the perceived effects of architectural barriers. Subgroup analyses of the NHIS-D may be vulnerable to errors resulting from non-response bias that occurred during the original survey. Cross-sectional designs limit inferences about causality. Time specific: longitudinal studies are required.
**Study: Tchalla 2012**		**Title: Efficacy of Simple Home-Based Technologies Combined with A Monitoring Assistive Centre in Decreasing Falls in a Frail Elderly Population (Results of the Esoppe Study)**			
**Authors: Achille Edem Tchalla, Florent Lachal, Noelle Cardinaud, Isabelle Saulnier, Devender Bhalla, Alain Roquejoffre, Vincent Rialle, Pierre-Marie Preux, Thierry Dantoine**					
Study type	Setting	Inclusion criteria	Definition of specific functional limitation	Exclusion criteria	Recruitment procedures
Longitudinal Perspective cohort (pilot study)	Correze district in Limousin area, Southwest France July 2009–June 2010	Frail older adults ≥65, registered on a list of frail elderly people and living at home	Fried frailty criteria ≥3 Functional autonomy Measure System Profile (ISO-SMAF) classification	People with a severe dementia: MMSE ≥25 People in a falls prevention rehabilitation programme	Participants were recruited through a population survey in Correze district (pre-selected by the council).
Samples	Interventions		Outcome measures	Results	Quality (MMAT) & Limitations
*N* = 194 (mean age 83.4 years, women 77.4%) *n* = 96 intervention group (mean age 84.9 years, women 76.6%) *n* = 98 control group (mean age 82.0 year, women 78.1%)	The intervention group received light path installed near the bed, which is a 1.5 m long and turns on automatically on when the person sets foot on the ground. The light path proved visibility by showing the right path and improving conscious awareness of environment. They also received tele-assistance service 24/7: a remote intercom, an electronic bracelet. The control group did not receive any intervention.		Incidence rate of fallsBaseline clinical assessment: medical history of previous falls, comorbidities and medications, ISO-SMAF classification, Tried Frailty criteria, MMSE, Mini Nutrition Assessment, Geriatric Depression Scale 12 months following inclusion in the study	After taking into account significant variables in the multivariate model, the use of light path coupled with tele-assistance was significantly associated with reduction in falls at home: OR = 0.33 95% CI = 0.17 to 0.65 *p* = 0.0012. There was a great reduction in post—fall hospitalisation rate in the intervention group: OR = 0.30 95% CI = 0.12 to 0.74 *p* = 0.0091.	MMAT ** Potential recall bias, especially in older adults population: this reporting bias can underestimate the rate of falls. Identification of the falls is influenced by knowledge of exposure group: over or under-estimation of falls.

RCT: randomised controlled trial; N/A: not applicable; MMAT: mixed method appraisal tool; MMAT *: * the lowest and **** the highest score; SD: standard deviation; CI: confidence interval; OR: odds ratio; ADL: activities of daily living; IADL: instrumental activities of daily living; CES-D: center for epidemiologic studies depression scale; NHIS-D: national health interview survey on disability; ISO-SMAF: functional autonomy measurement system.
